# Real-world adherence to toxicity management guidelines for immune checkpoint inhibitor-induced diabetes mellitus

**DOI:** 10.3389/fendo.2023.1213225

**Published:** 2023-07-24

**Authors:** Min Shen, Doudou Chen, Ruiling Zhao, Xuqin Zheng, Yong Gu, Tao Yang, Yun Shi

**Affiliations:** Department of Endocrinology and Metabolism, The First Affiliated Hospital of Nanjing Medical University, Nanjing, Jiangsu, China

**Keywords:** immune-related adverse events, immune checkpoint inhibitors, toxicity guideline adherence, diabetes mellitus, proposal

## Abstract

**Objective:**

Immune checkpoint inhibitors(ICIs) have improved survival and are increasingly used for cancer. However, ICIs use may be limited by immune-related adverse events (irAEs), such as ICI-induced diabetes mellitus(ICI-DM). The objective of the present study was to characterize ICI-DM patients and real-world adherence to guidelines.

**Research design and methods:**

The present study was a retrospective review of electronic records of ICI-DM patients at the First Affiliated Hospital of Nanjing Medical University between July 2018 and October 2022.

**Results:**

34.8% (8/23)patients monitored blood glucose in every treatment cycle. The proportion of patients with severe diabetic ketoacidosis(DKA) was lower in the tight glycemic monitoring group than the non-tight glycemic monitoring group (16.7% vs. 55.6%, *p* = 0.049). 78.3%(18/23) patients with hyperglycemia visited a non-endocrinologist first, but 95.7% of patients were then referred to an endocrinologist. Twenty patients were tested for distinguishing the etiology of hyperglycemia and 20% patients with positive glutamic acid decarboxylase antibody(GADA), 55% with C-peptide <3.33pmol/L. High screening rates for other ICI-induced endocrinopathies were observed and half of the patients with ICI-DM developed other endocrine gland irAEs, with the most common being thyroiditis. Moreover, five patients developed non-endocrine serious adverse events(SAEs). Twelve (52.2%) patients were withdrawn from ICI due to ICI-DM. The time to progression of tumor in ICI-DM patients in the continue and interruption group was longer than in the withdrawal group (333.5 ± 82.5 days vs. 183.1 ± 62.4 days, *p* = 0.161). Only 17.4% of ICI-DM patients were completely managed according to guidelines. Thus, the present study proposed a screening, diagnosis, and management algorithm for ICI-DM in real-world practice.

**Conclusion:**

The present study reported the largest number of ICI-DM cases described in a single institute, providing insight into real-world ICI-DM management guideline adherence and highlighting the clinical challenges in ICI-DM management.

## Introduction

Immunotherapy has revolutionized the treatment of many cancer types (1). Immune checkpoint inhibitors (ICIs) target the cytotoxic T lymphocyte antigen 4 (CTLA-4)/CD28/programmed cell death 1 (PD-1)/programmed cell death 1 ligand 1 (PD-L1) axis, leading to immune activation in the tumor microenvironment (2, 3). ICIs induce durable treatment responses in patients with advanced cancers; however, because ICIs activate T immune cells in a variety of tissues, they are often associated with autoimmune side effects, termed immune-related adverse events (irAEs) (4). These irAEs can affect almost any organ system and commonly affect the colon, liver, lungs, skin, and endocrine organs (3, 5). The irAEs tend to be mild and self-limited with low-grade effects (grades 1–2) in up to 90% of patients, while more severe effects (grades 3–5) are observed in 20–60% of patients (6–8).

Among the irAEs, ICI-induced endocrinopathies have been reported in up to 40% of patients, with thyroid disorders being the most common (2, 9–11). ICI-induced diabetes mellitus (ICI-DM) is a rare but potentially life-threatening complication that occurs in approximately 1% of patients receiving ICIs (12–16). ICI-DM is characterized by rapid β cell destruction (17, 18), and it has a more acute onset compared to the classic type 1 diabetes mellitus (2, 19). Most ICI-DM cases present as life-threatening diabetic ketoacidosis (DKA) or severe hyperglycemia, with symptom severity of grades 3–4 (18). Thus, early detection and management of ICI-DM are necessary to prevent significant morbidity and mortality.

To aid practicing oncologists, multiple guidelines for the evaluation and treatment of irAEs have been developed. Despite a lack of evidence on the optimal management of toxicities, several guidelines aim to aid clinicians in the detection and management of irAEs (20–28). While irAEs are well-characterized in clinical trials and observational studies, there are insufficient data on whether real-world management of these irAEs adheres to clinical practice guidelines. Most guidelines, including those utilized in the present study, are derived from clinical trial protocols and are not necessarily based on evidence.

Therefore, we characterized ICI-DM patients and their management in a real-world setting to identify opportunities for quality improvement and enhancement of patient care.

## Research design and methods

In this real-world retrospective study, patients were derived from the First Affiliated Hospital of Nanjing Medical University between July 2018 and October 2022. DM was diagnosed using the World Health Organization and American Diabetes Association criteria. ICI-DM was defined as new-onset insulin-dependent diabetes during ICIs treatment, characterized by an acute attack of severe hyperglycemia with the destruction of β cells and severe insulin deficiency (13, 29, 30). Hyperglycemia caused by radiotherapy, pancreatic surgery, pancreatic tumor, pancreatitis, glucose infusion and steroid were ruled out. The follow chart of our study process is illustrated in **Supplementary Figure 1**. The diagnostic criteria for fulminant type 1 diabetes mellitus were as follows: 1) occurrence of diabetic ketosis or ketoacidosis soon (approximately 7 days) after the onset of hyperglycemic symptoms (elevation of urinary and/or serum ketone bodies at the first visit); 2) plasma glucose level ≥ 16 mmol/L and hemoglobin A1c (HbA1c) level < 8.5% at the first visit; and 3) fasting serum C peptide level <0.3 or <0.5 ng/mL after intravenous glucagon loading or after a meal, respectively, at the first visit. DKA and its severity were classified according to the 2018 International Society for Pediatric and Adolescent Diabetes guidelines (31): mild DKA: venous pH < 7.3 or serum bicarbonate < 15 mmol/L; moderate: pH < 7.2, serum bicarbonate < 10 mmol/L; severe: pH < 7.1, serum bicarbonate < 5 mmol/L. The index for the glucose monitoring rate was calculated as the proportion of cycles with blood glucose tested.

Demographic, clinical, anthropometric, radiographic, and pathological data, including tumor type, ICIs regimen, and treatment response, for each subject were obtained from the review of the electronic medical record and telephone follow-up. For assessment of irAEs, we used the descriptions and grading scales of the National Cancer Institute Common Terminology Criteria for Adverse Events (version 5.0). We defined the use of immunosuppressive drugs as the use of traditional or biological disease-modifying antirheumatic drugs or glucocorticoids at supraphysiological doses for at least 30 days. In the present study, serious adverse events (SAEs) were defined as grade 3–5 irAEs. Body mass index (BMI) was calculated by dividing weight (kg) by height squared (m^2^). Age and BMI were extracted from the records at the initial diagnosis of ICI-DM. Tumor staging was histologically confirmed according to the 8th edition of the American Joint Committee on Cancer staging system before ICIs treatment. The best overall response of ICIs treatment was defined as complete response (CR), partial response (PR), stable disease (SD), or progressive disease (PD) documented on at least two consecutive imaging studies from the beginning of the ICIs regimen.

The present study was approved by the Ethics Committee of the First Affiliated Hospital of Nanjing Medical University (Jiangsu Province Hospital)

Patient and Public Involvement Statement:It was not appropriate or possible to involve patients or the public in the design, conduct, reporting, or dissemination plans of our research.

### Statistical analyses

The results are presented as counts with percentages for categorical variables and medians with interquartile ranges (IQRs) for continuous variables as these variables were not normally distributed based on the Kolmogorov–Smirnov test. Differences in the distribution of categorical variables were evaluated using the chi-square test or Fisher’s exact test. Continuous variables were compared using the Welch’s *t*-test. P < 0.05 was considered a statistically significant difference. All statistical analyses were performed using SPSS v25.0 (IBM Corp., Armonk, NY, USA), and the graphs were created using Prism v9.0.0 (GraphPad Software, Inc., La Jolla, CA, USA).

## Results

### Clinical characteristics of patients with ICI-DM

A total of 23 patients with ICI-DM were enrolled for analysis in the present study. The demographic and clinical data related to the malignancy are summarized in **Table 1**. Of the enrolled patients, 82.6% were males, and the average age was 61.84 ± 9.48 years. The majority of patients had a lean or normal body mass. In addition, 47.8% of patients had lung cancer, of whom 63.6% had non-small cell lung cancer. Digestive system cancer was the second most common tumor type followed by head and neck cancer. Among all patients, 91.3% were treated with PD-1 inhibitors, and the most widely used PD-1 inhibitors were tislelizumab (21.7%), toripalimab (17.4%), and camrelizumab (17.4%). One patient developed myocarditis after a single dose of toripalimab, and caused the ICIs treatment to be interrupted; corticosteroids treatment caused slight blood glucose fluctuations and positive glutamic acid decarboxylase antibody (GADA) of this patient was found. However, paianpulima (a novel PD-1 inhibitor) was reintroduced after improvement of the myocarditis and a significant decrease in the level of fasting C-peptide was observed (598.8 pmol/L to 221.1 pmol/L). Only two (8.7%) patients used durvalumab, a PD-L1 inhibitor. In addition, 17.4%(4/23) of patients were treated with radiotherapy before ICIs therapy, the radiotherapy sites were: colon, neck, lung and cardia of stomach; and 17.4%(4/23) were treated with radiotherapy during the ICIs therapy period, including 1 case of brain radiotherapy and 3 cases of lung radiotherapy. Five of the eight patients underwent pancreatic imaging and none of which showed significant abnormalities. Moreover, 2 (8.7%) patients were treated with chemotherapy before ICIs therapy, 4 (17.4%) were treated with an ICIs combined with chemotherapy, and 14 (60.9%) were treated with chemotherapy before ICIs therapy and then combined with ICIs. More than half of the patients underwent surgical removal of their primary tumor, and most tumors (91.3%) were histologically confirmed as stage III or IV.

**Table 1:** Baseline characteristics of study participants.

Characteristic	Value	%
**Number of participants (n)**	23	
Sex (n)		
Male	19	82.6%
Female	4	17.4%
**Age (years)**	61.84 ± 9.48	—
**BMI (kg/m^2^)**	21.43 ± 3.39	—
Tumor type (n)		
Head and neck cancer	3	13.0%
Lung cancer	11	47.8%
SCLC	4	36.4%
NSCLC	7	63.6%
Digestive system cancer	6	26.1%
Renal cancer	1	4.3%
Lymphoma	1	4.3%
Melanoma	1	4.3%
Causative agent(n)		
Anti-PD-1		
Nivolumab	1	4.3%
Pembrolizumab	3	13.0%
Sintilimab	3	13.0%
Toripalimab	4	17.4%
Camrelizumab	4	17.4%
Tislelizumab	5	21.7%
Toripalimab + Penpulimab	1	4.3%
Anti-PD-L1		
Durvalumab	2	8.7%
Radiotherapy(n)		
No	15	65.2%
Before ICIs	4	17.4%
Combined with ICIs	4	17.4%
Chemotherapy(n)		
No	3	13.0%
Before ICIs	2	8.7%
Combined with ICIs	4	17.4%
Before and combined with ICsI	14	60.9%
Surgical treatment(n)		
No	10	43.5%
Yes	13	56.5%
Tumor staging (Before ICIs)(n)		
II	2	8.7%
III	7	30.4%
IV	11	47.8%

BMI, body mass index; SCLC, small cell lung cancer; NSCLC, non-small cell lung cancer; ICIs, immune checkpoint inhibitors.

As shown in **Table 2**, the median time to ICI-DM diagnosis from the initiation of ICIs treatment was 10 (IQR: 5–12) cycles and 44.1 (IQR: 18.4–52.3) weeks, 16 (69.6%) patients were diagnosed with ICI-DM after 6 months ICIs treatment. Moreover, 65.2% (15/23) of cases presented with DKA. The median plasma glucose level at diagnosis was 28.6 (IQR: 18.5–66.3) mmol/L with an elevated HbA1c level (8.5%, IQR: 7.5–10.0) and a low fasting C-peptide level (8.62 pmol/L, IQR: 3.33–46.05). Fulminant type 1 diabetes mellitus was diagnosed in 14 (60.9%) patients. Increased plasma amylase level was found in 18.2% (2/11) of cases. Notably, two patients had preexisting diabetes before ICIs therapy, including one patient with type 2 diabetes mellitus and the other with latent autoimmune diabetes in adults. Besides, two patients had impaired fasting glucose before ICIs therapy.

**Table 2:** Characteristics of patients with ICI-DM.

Characteristic	Value
Interval to onset after ICIs therapy initiation	
Number of cycles of therapy, n	10 (5–12)
Duration to onset, weeks	44.1 (18.4–52.3)
**Glucose at diagnosis, mmol/L**	28.6 (18.5–33.3)
**HbA1c at diagnosis, %**	8.5 (7.5–10.0)
CTCAE level at diagnosis(n)	
**II**	3
**III**	8
**IV**	12
**DKA (n, yes/no)**	15/8
**C-peptide at diagnosis, pmol/L**	8.62 (3.33–46.05)
**Fulminant type 1 diabetes mellitus (n, yes/no)**	14/9
β-cell antibodies, n positive/n tested (%)	
GADA^*^	4/1/20
ICA	0/20
IA-2A	0/20
IAA	0/20
**Increased amylase (n, yes/no/untested)**	2/9/12
**History of DM (n, yes/no)**	2/21
**Best response of ICIs treatment(n, CR/PR/SD/PD)**	1/5/14/3
Combined other endocrine glands irAE(n)	
Thyroid	8
Hypophysitis	3
Thyroid + Adrenal gland	1
Thyroid + Hypophysitis	1
Combined non-endocrine SAE (n)	
Myocarditis	1
AKI	1
AKI, AMI	1
Cerebral demyelinating lesions	1
Dermatological AE	1
**Treatment interrupt/withdrawal/continue (n)**	3/12/8

^*^ Four cases were positive for GADAs, and one case had uncertain GADA positivity.

IAA, insulin antibody; IA-2A, insulinoma-associated protein 2 antibody; GADA, glutamic acid decarboxylase antibody; ICA, islet cell autoantibody; CR, complete response; PR, partial response; SD, stable disease; PD, progressive disease; DKA, diabetic ketoacidosis; HbA1c, hemoglobin A1c; CTCAE, Common Terminology Criteria for Adverse Events; irAE, immune-related adverse event; SAE, serious adverse event; AKI, acute kidney injury; AMI, acute myocardial infarction.

After ICI-DM diagnosis, 47.8% (11/23) of patients continued taking the ICI therapy. The objective response rate (complete response and partial response) of ICIs treatment was 26.1% (6/23) and 60.9% (14/23) were stable. Half of the patients with ICI-DM developed other endocrine gland irAEs, with the most common being thyroiditis. Moreover, five (21.7%) patients developed non-endocrine serious adverse events(SAEs), including myocarditis, acute kidney injury, acute myocardial infarction, cerebral demyelinating lesions, and dermatological adverse events.

### Monitoring symptoms and glucose level at treatment initiation and during treatment cycles

There was insufficient monitoring of the clinical signs and symptoms. None of the patients reported that they were asked about the development of the polyuria-polydipsia syndrome, loss of weight, or clinical signs of ketoacidosis before ICIs treatment and during each treatment cycle.

**Figure 1:**
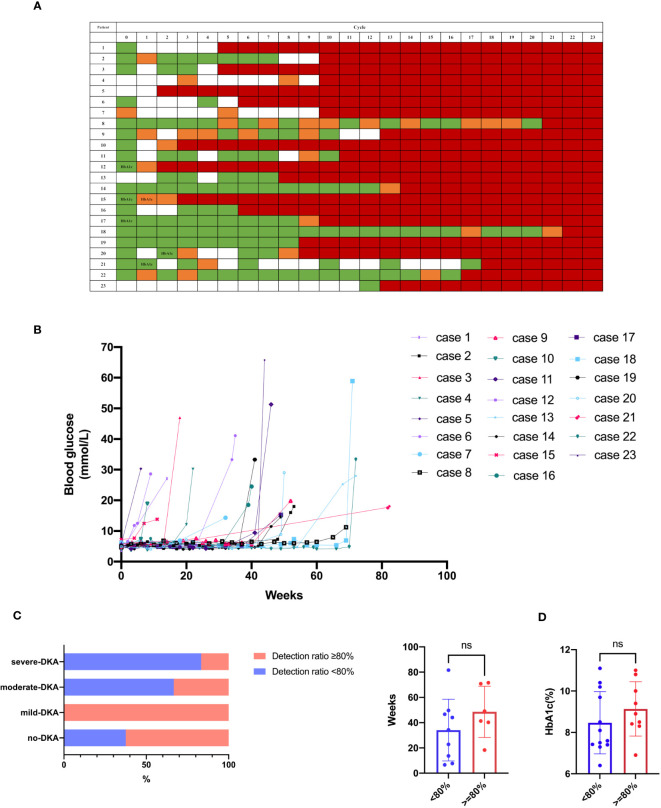
Blood glucose monitoring. **(A, B)** Glucose level during ICIs treatment for each case. Green indicates normoglycemia, and orange indicates hyperglycemia (> 6.1 mmol/L) prior to clinical diagnosis. White indicates an undetected case, and red indicates clinical diagnosis. HbA1c indicates HbA1c test prior to clinical diagnosis. **(C)** DKA occurrence between the groups of blood glucose monitoring rate ≥ 80% and < 80%. **(D)** HbA1c between the groups of blood glucose monitoring rate ≥ 80% and < 80%. ns, not significant.

We retrospectively reviewed the glucose level measured during the ICIs treatment for each patient (**Figures 1A, B**). The frequency of blood glucose monitoring varied among these patients. Thirteen patients without a history of diabetes had incidental finding of mild hyperglycemia during ICIs therapy prior to the clinical diagnosis; however, physicians did not increase the surveillance frequency or perform further evaluation. The blood glucose monitoring rate after the detection of hyperglycemia was 74% (31–100%). Moreover, glucose was measured in every treatment cycle for 8 (34.8%) patients. The patients were divided into two groups according to their blood glucose monitoring rate as follows: 11 patients had a blood glucose monitoring rate ≥ 80% (tight monitoring), and 12 patients had a blood glucose monitoring rate < 80% (non-tight monitoring). There was no statistical difference in the proportion of patients with DKA between these two groups (54.5% vs. 75%, respectively, *p* = 0.400) (left panel of **Figure 1C**). However, the proportion of severe DKA was lower in the tight monitoring group than the non-tight monitoring group (16.7% vs. 55.6%, respectively, *p* = 0.049). No significant difference was found in the time from DKA occurrence to the initiation of ICIs treatment between the two groups (*p* = 0.252) (right panel of **Figure 1C**). Moreover, the HbA1c level did not differ between the two groups (*p* = 0.285) (**Figure 1D**). Only two patients underwent testing for the glycated albumin (GA) level at diagnosis, with values of 22.8% and 24.8%.

### Diagnosis and etiology of hyperglycemia

**Figure 2:**
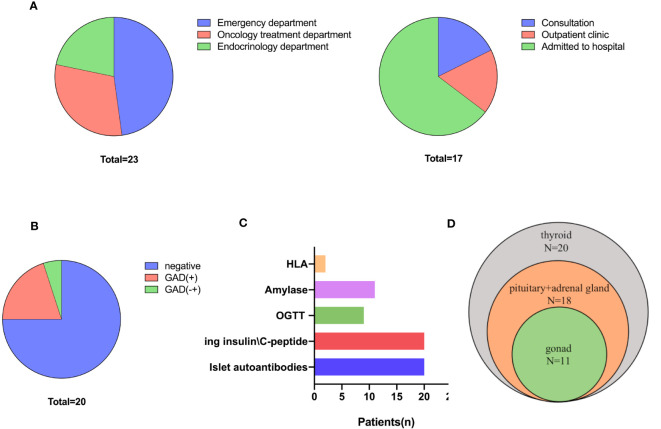
Diagnosis and classification of ICI-DM. **(A)** First-visit (left) department and referral department. **(B)** β cell antibodies. **(C)** Screening and diagnosis of ICI-DM. **(D)** Screening for other endocrine disorders.

There were high referral and multidisciplinary rates. When diagnosed with hyperglycemia, 78.3% of patients visited a non-endocrinologist first, but these patients, except for one, were then referred to the Department of Endocrinology. Among the referred patients, 3 received endocrinology consultation, 3 visited the endocrinology outpatient clinic, and 11 were hospitalized in the Department of Endocrinology. Of the five patients who first visited the Department of Endocrinology, 80% were admitted to the hospital (**Figure 2A**).

There were high diagnosis and differential diagnosis rates. Twenty (87.0%) patients were tested for β cell antibodies and fasting insulin/C-peptide levels; nine of these patients underwent an oral glucose tolerance test. In addition, 20% (4/20) of cases were positive for glutamic acid decarboxylase antibody (GADA), and 1 had uncertain GADA positivity (**Figures 2B, C**). No other β cell antibodies (insulin autoantibody, IAA; islet cell antibody, ICA; and insulinoma-associated protein 2 antibody, IA-2A) were positive. The C-peptide level was <3.33 pmol/L in 55% (11/20) of patients, whereas the C-peptide level was declined in 11 patients (**Figure S2**). Two patients were tested for human leukocyte antigen (HLA) genes and were found to have the following susceptible HLA haplotype for type 1 diabetes mellitus: HLA‐A*1101:2402‐DRB1*0301:0301 and HLA-DRB1*0405;1101-HLA-DQB1*0301/27;0401 (**Figure 2C**).

### Screening rates for other ICI-endocrinopathies

The present study indicated that there were high screening rates for other ICI-induced endocrinopathies. Only two (8.7%) patients were not screened for other endocrine disorders due to the high endocrinology referral rate. All other patients were tested for thyroid function, and abnormal thyroid function was identified in half of the patients and one was central hypothyroidism. In addition, 18 (78.3%) patients were tested for hypothalamic-pituitary-adrenal axis function, of whom 3 were found to have abnormalities, and physiological replacements of glucocorticoids were used to treat. Eleven (47.8%) patients were screened for hypothalamic-pituitary-gonad axis function and 1 was found to have abnormalities (**Figure 2D**).

### Treatment for patients with ICI-DM

**Figure 3:**
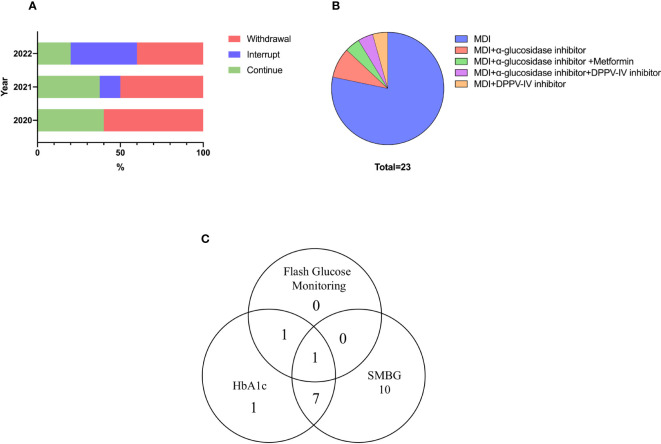
Treatment and blood glucose monitoring measures for ICI-DM. **(A)** Choice of ICIs treatment. **(B)** Glucose-lowering drugs. **(C)** Glucose monitoring measures.

None of the patients used high-dose glucocorticoids or immunosuppressants to treat diabetes. Half of the patients discontinued the ICIs treatment due to ICI-DM. After the occurrence of ICI-DM, ICIs treatment was interrupted in three (13.0%) patients until good glycemic control was achieved. Twelve (52.2%) patients discontinued ICIs due to ICI-DM, but one of them restarted another ICIs regimen after reevaluating tumor progression. In addition, eight (34.8%) patients continued to use the ICIs regimen. From 2020 to 2022, the proportion of patients who were withdrawn from ICIs treatment gradually decreased, but this trend did not reach significance (**Figure 3A**). Only one of the patients who did not withdraw from ICIs had SAEs (cerebral demyelinating lesions) during subsequent ICIs treatment. The time to progression of tumor in ICI-DM patients in the continued and interrupted treatment groups was longer than in the withdrawal group (333.5 ± 82.5 days vs. 183.1 ± 62.4 days, respectively, *p* = 0.161), but there was no statistical significance due to the small sample size. However, the trend suggested clinical significance.

Nearly all patients received multiple daily insulin regimens. Twenty-two (95.7%) patients received insulin doses four times a day until the last visit, while only one patient used premixed insulin. Daily insulin dose were 0.45(IQR: 0.39–0.66) IU/kg/d. In addition, 21.7% of patients used insulin therapy combined with oral hypoglycemic drugs. The most common oral medications were α-glucosidase inhibitors and dipeptidyl-peptidase IV (DPP-IV) inhibitors (**Figure 3B**).

### Follow-up visit for ICI-DM

Patients were followed up for the longest period of 4 years and the shortest period of 3 months, and five patients passed away due to tumor progression by October 2022. Fifteen (65.2%) cases presented to the Department of Endocrinology with diabetes-related problems, and the other cases presented to the treatment-related departments, except for two who were lost to follow-up. The most common reasons for patient presentation were review and treatment for tumor, medication prescription, and large glucose fluctuations.

Most patients chose self-monitoring of blood glucose for glucose measurements, and 83.3% of these patients monitored blood glucose daily, with the remaining patients monitoring glucose irregularly. Six patients underwent HbA1c level measurement every 3 months, two underwent testing every month and two underwent testing irregularly. The self-reported standard-reaching rate of HbA1c (< 8.5%) was 70%. Flash glucose monitoring, a novel glucose measurement technique, was used by two patients (**Figure 3C**).

Only three patients reported being asked for symptomatic and asymptomatic hypoglycemia at each visit, while eight patients reported one or more episodes of hypoglycemia. However, only two of these patients presented to the Department of Endocrinology to reevaluate the insulin protocol. Chronic complications of diabetes, including diabetic nephropathy, retinopathy, and macroangiopathy, were assessed in 11 (47.8%) patients, but none of these patients had these complications. Thus, these findings indicated low rates of chronic complications of diabetes.

### Review of the guidelines and expert consensus for ICI-DM

We reviewed several guidelines and consensus as well as their updates from the Society for Immunotherapy of Cancer (SITC), European Society for Medical Oncology (ESMO), American Society of Clinical Oncology (ASCO), National Comprehensive Cancer Network (NCCN), French Endocrine Society, and Chinese Society of Endocrinology. **Table 3** shows the summary of the methods used for ICI-DM monitoring, screening, diagnosis, and management. As expected, there were some inconsistencies, such as when and how to monitor the glucose level, islet antibody testing order, and type of antibody panel used.

**Table 3:** Review of the guidelines or expert consensuses for ICI-DM.

Guideline	Monitoring		Distinguish		ICIs treatment
	Monitor symptoms	Monitor glucose	Islet function	β-cell antibodies	
Society for Immunotherapy of Cancer (SITC)					
**SITC** **(2017**) **(** 26 **)**	• Monitor symptoms • Patient education	• Glucose and HbA1c levels at baseline; • Metabolic panel before each cycle;	• C-peptide • Insulin	• GADA • IAA • ICA • ZnT8A	• Without DKA: hold ICIs for grade≥3, and continue ICIs when recover to grade 1; • With DKA: hold ICIs
**SITC** **(2021**) **(** 25 **)**	• No emphasis	• CMP before and throughout the ICsI therapy. • HbA1c for elevated glucose	• C-peptide	• Ab	• Hold ICIs until DKA is resolved
European Society for Medical Oncology (ESMO)					
**ESMO** (2017) (20 **)**	• No emphasis	• Glucose level at baseline Regular monitoring of glucose levels*	• C-peptide	• GADA • ICA	• Consider restarting treatment with ICIs once the patient has been regulated with insulin substitution;
**ESMO** (2022) (21 **)**	• No emphasis	• Glucose and/or HbA1c every 4–6 weeks or every cycle. • Repeat HbA1c if suspected T1DM	• C-peptide	• GADA • IAA	• Asymptomatic or mild symptoms: Continue ICIs therapy with close follow-up; • Moderate symptoms and no DKA: hold ICIs therapy until stabilized; • Severe or life-threatening symptoms, ketoacidosis: hold ICIs therapy until stabilized
American Society of Clinical Oncology (ASCO)					
**ASCO** (2018) (23 **)**	• Monitor symptoms	• Glucose at baseline and with each treatment cycle during induction for 12 weeks, then every 3–6 weeks thereafter	• C-peptide • Insulin	• GADA • IAA • ICA	• Patients with grade≥3 should hold ICIs until glucose control is obtained on therapy with reduction of toxicity to grade 1 or less
**ASCO** (2021) (24 **)**	• Monitor symptoms	• Glucose at baseline and with each treatment cycle while on therapy and at follow-up visits for at least 6 months.	• C-peptide	• GADA • ICA	• Patients with grade≥3 should hold ICIs until glucose control is obtained on therapy with reduction of toxicity to grade 1 or less
National Comprehensive Cancer Network (NCCN)					
**NCCN** (2019) (27 **)**	• Monitor symptoms	• CMP at baseline and repeat prior to each treatment or every 2–3 weeks during immunotherapy then in 6–12 weeks. • HbA1c for elevated glucose	• C- peptide	• GADA • ICA	• Without DKA: continue ICIs; • With DKA: hold ICIs
**NCCN** (2020) (28 **)**	• Monitor symptoms	• CMP at baseline and repeat prior to each treatment or every 4 weeks during immunotherapy then in 6–12 weeks. • HbA1c for elevated glucose	• C-peptide	• GADA • ICA	• Without DKA: continue ICIs; • With DKA: hold ICIs
French Endocrine Society					
**French Endocrine Society** (2019) (22 **)**	• Patient education	• Fasting venous blood glucose (only with anti-PD-1/PD-L1) at each appointment during the first 6 months, and at every second appointment over the following 6 months, then on appearance of clinical signs. • HbA1c for elevated glucose	• No emphasis	• GADA first • if absent, testing for IA2A and ZnT8A	• Development of diabetes during ICIs with anti-PD-1 or PD-L1 is not a contraindication to continued ICIs use. • Where the situation is severe, ICsI can be delayed for a few days.
Immune-endocrinology Group, Chinese Society of Endocrinology, Chinese Medical Association					
**Chinese Society of Endocrinology** (2020)	• No emphasis	• Glucose at baseline and with each treatment cycle and at follow-up visits for every 3–6 weeks	• OGTT	• GADA first • if absent, testing for IA2A and ZnT8A	• Patients with grade ≥ 2 toxicity should hold ICIs until glucose control is obtained on therapy with reduction of toxicity to grade 1 or less

CMP, comprehensive metabolic panel; OGTT, oral glucose tolerance test; T1DM, type 1 diabetes mellitus.

*For anti-CTLA4 treatment, monitoring should occur every 3 weeks prior to every infusion during the first 12 weeks; thereafter, monitoring should occur at every follow-up visit (preferably every 6 weeks) for a period of 3 months after the last treatment and every 3 months thereafter. For anti-PD-1/PD-L1 treatment, monitoring should occur at 2 weeks, every 2 weeks during the first 12 weeks, and, in the case that these are normal, every 4 weeks and until 3 months after last anti-PD-1/PD-L1 infusion at every follow-up visit; thereafter, monitoring should occur every 3 months. In the case of monitoring every 3 weeks for the anti-PD-1/PD-L1 infusion, lab tests should be performed before every infusion and at least until 3 months after the last infusion at every follow-up visit; thereafter, monitoring should occur every 3 months. For the combination of anti-CTLA4 + anti-PD-1/PD-L1, lab tests should be performed at every infusion (also in period of anti-PD-1/PD-L1 maintenance) and every 6 weeks until 3 months after the last infusion; thereafter, monitoring should occur every 3 month.

### Proposed screening and treatment algorithm for ICI-DM

Despite following irAEs management guidelines, only 17.4% (4/23) of patients were completely managed according to guidelines and none of the patients reported to be asked about their clinical signs or symptoms. Only 34.8% of patients underwent blood glucose monitoring at every ICI cycle, and 47.8% continued ICIs treatment after hyperglycemia. In contrast, 87% of patients were tested for the C-peptide level and β cell antibodies, and 91.3% of patients were screened for other endocrine disorders. Moreover, all patients received multiple doses of insulin daily.

**Figure 4:**
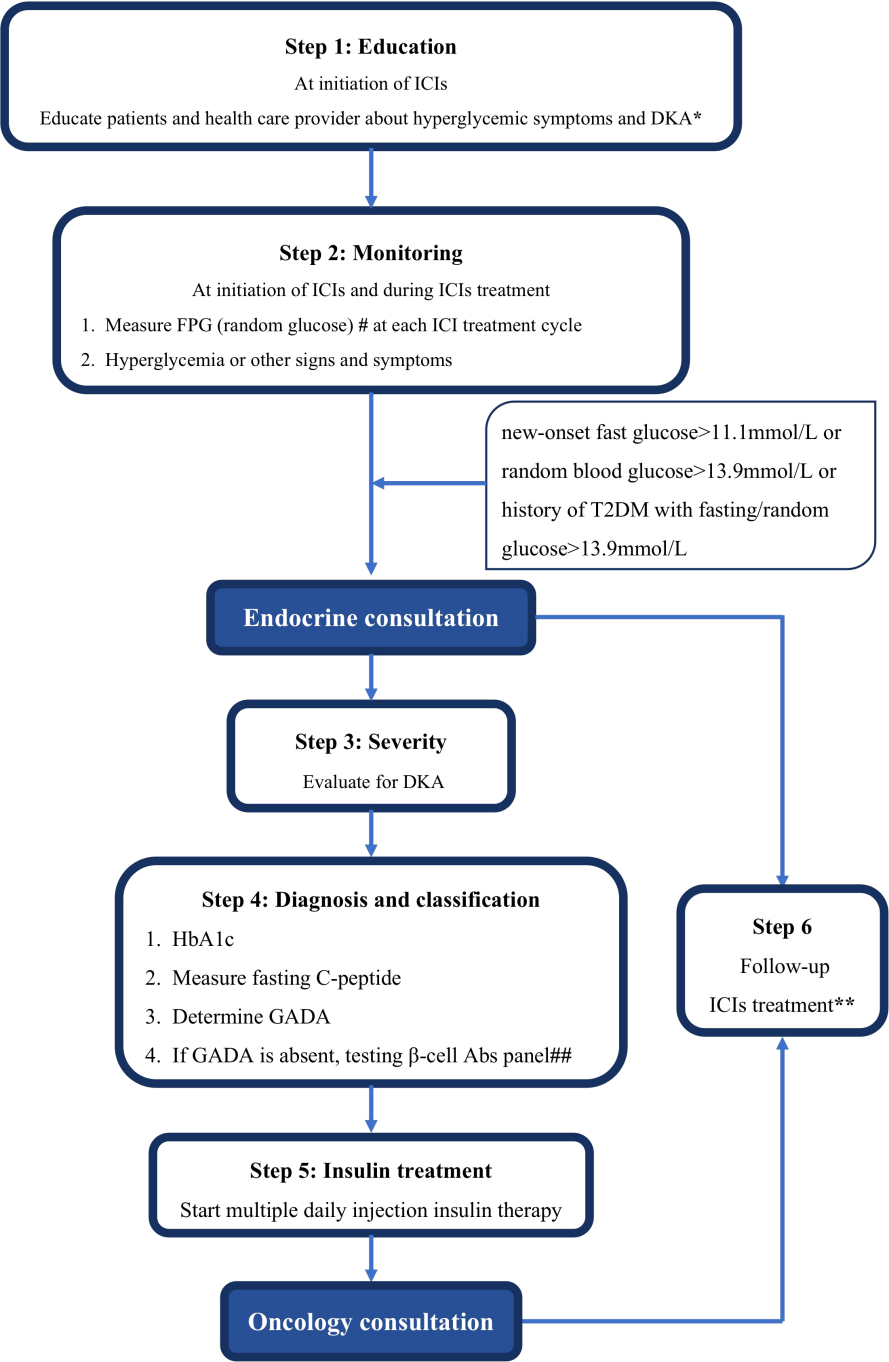
Proposal of screening, diagnosis, and management algorithm for ICI-DM in patients treated with ICIs. *, polyuria-polydipsia syndrome, weight loss, or clinical signs evoking ketoacidosis, symptoms of diabetic ketoacidosis may include excessive thirst, frequent urination, general weakness, vomiting, confusion, abdominal pain, dry skin, dry mouth, increased heart rate, and fruity odor on the breath; ^#^=fasting glucose preferred; and ^##^=IAA, ICA, IA-2A, and ZnT8A; **Patients with grades 3–4 toxicity should hold ICIs treatment until glucose control is obtained on therapy, with reduction of the severity of toxicity to grade 1 or less. It is not recommended of permanent discontinuation of ICIs treatment due to ICI-DM. Patients should then be referred to oncologists to decide whether or not to resume ICIs treatment.

As immunotherapies are increasingly used, the incidence of ICI-DM will gradually increase. Clinicians should be aware of irAEs, including ICI-DM and other endocrinopathies. In clinical practice, we propose to educate patients and health providers about hyperglycemic symptoms and DKA at the time of initiation of ICIs. In all patients, fasting or random plasma glucose (fasting glucose preferred) should be monitored at each cycle of ICI therapy as well as when patients are hyperglycemic or have other signs or symptoms. Patients with new-onset hyperglycemia (new-onset fasting glucose level > 11.1 mmol/L, random blood glucose level > 13.9 mmol/L, or history of type 2 diabetes mellitus with fasting/random glucose level > 13.9 mmol/L) should be referred to endocrinologists for endocrine consultation. A basic metabolic panel, acidemic pH, urine ketones, serum ketones, HbA1c, fasting C-peptide, and GADA should be tested. If GADA is absent, a β cell antibody panel (insulin autoantibody, IAA; islet cell antibody, ICA; insulinoma-associated protein 2 antibody, IA-2A and zinc transporter 8 autoantibody, ZnT8A) should be tested. These measurements will help to diagnose DKA and distinguish the etiology of hyperglycemia when other causes are suspected, such as type 2 diabetes mellitus, stress hyperglycemia, or steroid-induced hyperglycemia. If DKA is confirmed, it should be managed according to institutional guidelines. Once ICI-DM is diagnosed, patients should receive multiple daily insulin injections (basal-bolus regimen). Patients with grades 3–4 toxicity should hold ICIs treatment until glucose control is obtained on therapy, with reduction of the severity of toxicity to grade 1 or less. It is not recommended of permanent discontinuation of ICIs treatment due to ICI DM. Patients should then be referred to oncologists to decide whether or not to resume ICIs treatment. Overall, the management of ICI-DM should be directed by specialized multidisciplinary teams, namely endocrinology and oncology (**Figure 4**).

## Discussion

To the best of our knowledge, the present study reported the largest number of cases with ICI-DM described in a single institute. This was the first study to include guideline adherence as an outcome and to report the proportion of irAEs that were managed according to guidelines in a real-world setting. The present study provided insight into real-world irAEs management guideline adherence with 82.6% of ICI-DM patients not managed according to guidelines. We highlighted the clinical challenges in the management of patients with ICI-DM and proposed a screening, diagnosis, and management algorithm for ICI-DM in the real-world setting.

ICIs profoundly affect oncologic care and result in immune-mediated antitumor activity. However, normal tissues may be affected, leading to irAEs (2). ICI-DM is a rare irAE with previously reported rates of approximately 1% (12–16). The rapid development of DM as a consequence of PD-1/PD-L1 inhibition is the result of acute loss of β cells, which manifests as rapid transition from normoglycemia to hyperglycemia, and it may be irreversible (15, 17). The hyperglycemia in ICI-DM is correlated with a mild increase in HbA1c level (17). As shown in the present and previous studies, the most recent random blood glucose levels measured within weeks before ICI-DM are predominantly normal or only mildly elevated (32). Thus, recognizing ICI-DM and initiating appropriate treatment are crucial components of drug monitoring in cancer patients treated with ICIs.

Guidelines for irAEs are rapidly evolving and becoming more site-specific with the involvement of expert subspecialists. Most oncology guidelines, including those utilized in the present study, are derived from clinical trial protocols and are not necessarily based on evidence. Multiple guidelines have been developed that outline how to screen, diagnose, and manage these endocrine toxicities (20–28). We found that less than 20% of patients with ICI-DM were managed according to guidelines. The poor guideline adherence in real-world clinical practice mainly included low blood glucose monitoring rate, β cell antibody testing order, antibody panel used, and determination of appropriate ICIs treatment withdrawal.

First, most of the guidelines or expert consensuses recommend that blood glucose levels should be regularly monitored in patients treated with ICIs to detect the emergence of diabetes mellitus. In patients treated with anti-PD-1 or anti-PD-L1, however, it is recommended that the appearance of polyuria-polydipsia syndrome, weight loss, or clinical signs evoking ketoacidosis should lead to immediate testing of blood glucose level according to the French Endocrine Society guidance on endocrine side effects of immunotherapy (2019) (22). According to the ASCO clinical practice guideline (2018), patients should be monitored for hyperglycemia or other signs and symptoms of new or worsening diabetes mellitus, including measuring glucose level at baseline, with each treatment cycle during induction for 12 weeks, and every 3–6 weeks thereafter (23). The ASCO clinical practice guideline (2021) recommends that patients should be monitored during each treatment cycle while on therapy and at follow-up visits for at least 6 months (24). Previous studies have shown that symptoms typically present within 6 months of ICIs initiation, but the time of onset is unpredictable and may arise any time on therapy or even several months after ICIs cessation (12). The severity of these events also varies widely. In the present study, we found that few clinicians monitored patients for symptoms of new or worsening diabetes mellitus, such as polyuria, polydipsia, and fatigue. We also observed a significant difference in blood glucose monitoring frequency. The patients with tight monitoring in nearly every treatment cycle (≥ 80%) had a lower proportion of severe DKA than other patients. Moreover, 16 (69.6%) patients were diagnosed with ICI-DM after 6 months. Because ICI-DM generally manifests as an insulin-dependent diabetes mellitus, it is also possible that exacerbation of underlying type 2 diabetes mellitus or latent autoimmune diabetes in adults remains unrecognized. In the present study, two patients (one with type 2 diabetes mellitus and the other with latent autoimmune diabetes in adults) experienced increased blood glucose levels and were diagnosed as ICI-DM. Thus, monitoring glucose levels and symptoms of patients at baseline and with each treatment cycle during ICIs treatment is critical in the real-world setting.

Second, it is recommended that C-peptide levels and β cell antibodies should be measured to distinguish between type 1 and type 2 diabetes mellitus. Previous data have suggested that ICI-DM is characterized by rapid β cell destruction. Therefore, measurement of C-peptide levels is a useful tool for the diagnosis of ICI-DM, and its evaluation should be performed more than once to detect the evolution of β cell function. In the present study, 87.0% of patients were tested for β cell antibodies and fasting insulin/C-peptide levels when the patients were diagnosed with diabetes mellitus. Although antibody testing is recommended to distinguish between type 1 and type 2 diabetes, there was inconsistency in the islet antibody testing order and type of antibody panel used. As recommended by the French Endocrine Society (2019) (22), testing for GADA should be performed first; if these are absent, testing for insulinoma-associated protein 2 antibody (IA-2A) and anti-ZnT8 antibodies (ZnT8A) should be performed. Tests for GADA, IAA, ICA, and ZnT8A are recommended by the SITC (2017) (26). GADA and ICA should be measured to distinguish between type 1 and type 2 diabetes mellitus according to ESMO (2017) (20). GADA, ICA, and IAA are highly specific for autoimmune diabetes according to the ASCO (2018) (23), whereas GADA and ICA is recommended by the ASCO (2021) (24) and NCCN (2019) (27). In the present study, only 20% of patients were positive only for GADA and one patient with uncertain GADA positivity, which was consistent with a previous study that suggested that less than 50% of patients are positive for islet antibodies, with GADA being the predominant antibody. Thus, to diagnose and distinguish ICI-DM, we recommend that fasting C-peptide and GADA levels should be measured first; if GADA is absent, an antibody panel, including IA-2A, ZnT8A, ICA, and IAA, should be performed.

Third, nearly all guidelines recommend that when patients are diagnosed with ICI-DM, immunotherapy can be continued in patients with mildly elevated glucose, but ICIs should be held until glucose control is obtained on therapy with reduction of severity of toxicity to grade 1 or less in patients with higher glucose levels. The time to interruption of ICIs and when to restart ICIs vary between guidelines. According to the ASCO clinical practice guidelines (2018 and 2021) (23, 24), patients with grades 3–4 toxicity should hold ICIs until glucose control is obtained on therapy with reduction of severity of toxicity to grade 1 or less. According to the SITC (2017) (26), type 1 diabetes patients without DKA should stop ICIs treatment when hyperglycemia ≥ grade 3 occurs and should be treated with insulin until recovery to grade 1 is achieved. Subsequently, ICIs treatment can be continued. In addition, type 1 diabetes patients with DKA should stop ICIs treatment. According to the NCCN (2019) (27), patients without DKA should continue immunotherapy, and those with DKA should stop immunotherapy. Nevertheless, some guidelines are not clear and definite, such as the French Endocrine Society guidance on endocrine side effects of immunotherapy (2019) (22). The French Endocrine Society suggests that the development of diabetes during ICIs treatment with anti-PD-1 or PD-L1 is not a contraindication for continued ICsI, but when the situation is severe, ICIs treatment can be delayed for a few days. Despite the irAEs management guidelines, the present study demonstrated that half of patients were withdrawn from ICIs treatment due to ICI-DM, and that the proportion that was withdrawn from ICIs treatment decreased yearly. Moreover, we found that the time to progression of tumor from ICI-DM in the continued and interrupted treatment groups was longer than that in the withdrawal group; however, there were no significant increases in SAEs in patients who did not withdraw the ICIs.

The major strengths of the present study were the inclusion of guideline adherence as an outcome and the proportion of ICI-DM patients that were managed according to guidelines in the real-world setting. We obtained all parameters, both clinical and laboratory, to characterize the ICI-DM in the real world and determine the features leading to poor guideline adherence. By characterizing these events and their management in a real-world setting, opportunities for quality improvement and enhancement of patient care were identified. Therefore, based on previous guidelines, we propose an algorithm for screening, diagnosis, and management of ICI-DM in the real-world setting. However, the present study had several limitations. First, the present study was a single-center retrospective study with a relatively small sample size, which lacked sufficient power to detect significant subgroup differences. Second, lack of long-term follow-up and prospective data made it difficult to assess the impact of ICI-DM to therapeutic effects of tumor. Third, because the low incidence of ICI-DM as an adverse effect of PD-1/PD-L1 inhibition makes it difficult to study the patient cohort at risk for developing such complications, further studies, including a large number of cases, are needed. Thus, we suggest that a registry for case reports involving adverse effects of PD-1/PD-L1 inhibitors should be established to allow a more comprehensive review.

## Conclusion

As the use of immunotherapies becomes prevalent, ICI-DM cases will gradually increase. ICI-DM is rarely fatal, but it can have notable effects on the quality of life of patients. Management of ICI-DM remains a key objective for practicing endocrinologists, who require skills to successfully diagnose and manage ICI-DM. Thus, it is critical to understand the presentation, potentially life-threatening nature, and natural history of ICI-DM. Although multiple guidelines for evaluation and treatment have been developed, poor guideline adherence is observed in the real-world setting. Therefore, by assessing the adherence to clinical guidelines for screening, diagnosis, and management of ICI-DM in real-world practice, we identified the potential problems and proposed a screening and treatment algorithm for ICI-DM.

## Data availability statement

The original contributions presented in the study are included in the article/**Supplementary Material**. Further inquiries can be directed to the corresponding authors.

## Ethics statement

The studies involving human participants were reviewed and approved by the Ethics Committee of the First Affiliated Hospital of Nanjing Medical University (Jiangsu Province Hospital). The patients/participants provided their written informed consent to participate in this study.

## Author contributions

TY and YS initiated and led the project. MS, DC, RZ and YG collected and analyzed the data. XZ and YS reviewed and edited the article. All authors contributed to the article and approved the submitted version. YS and TY are responsible for the integrity of the work as a whole. All authors approved the final version of the manuscript.

## Supplementary material

Supplementary Figure 1 The follow chart of this study process.

Supplementary Figure 2 Follow-up of 11 patients’ C-peptide. Visit 1: First C-peptide test due to elevated blood glucose during ICIs treatment. The interval between each visit varied among different patients.
