# Supplementary figures and images for: Real-world adherence to toxicity management guidelines for immune checkpoint inhibitor-induced diabetes mellitus
Source: Front Endocrinol (Lausanne). 2023 Jul 24;14:1213225. doi: 10.3389/fendo.2023.1213225 (PMC10405819; doi:10.3389/fendo.2023.1213225)

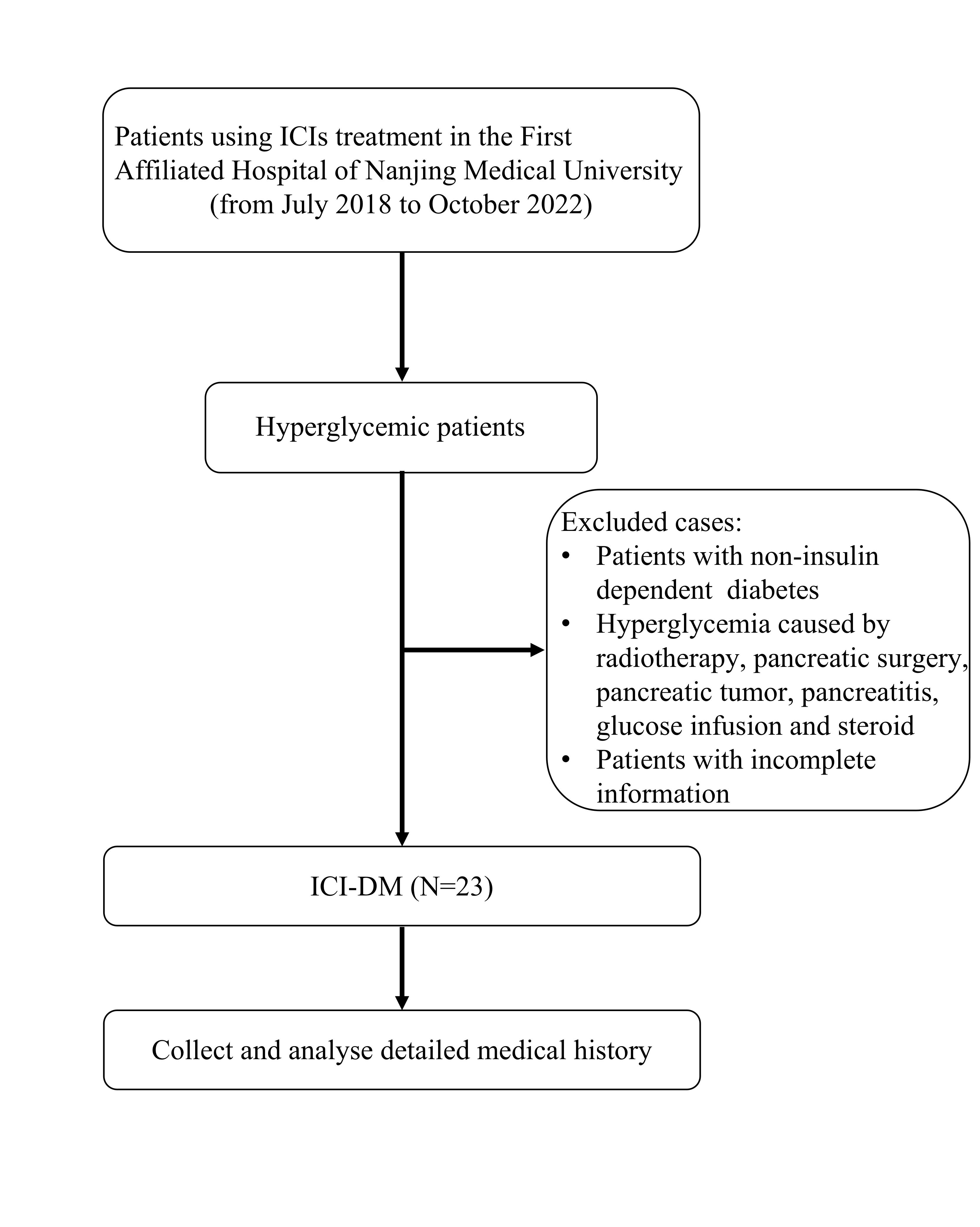

Supplement: Supplementary Figure 1 — The follow chart of this study process. [file Image_1.jpeg]

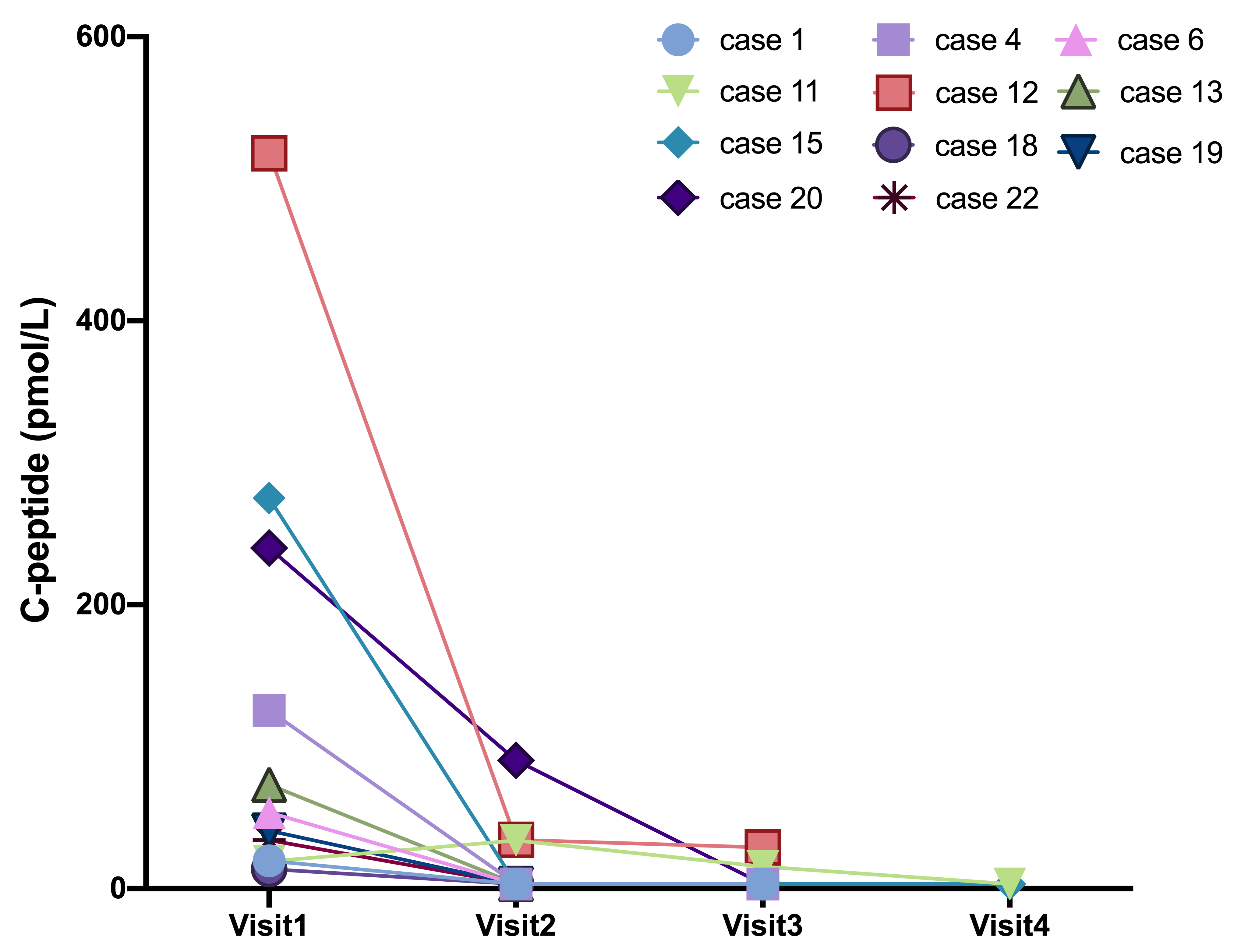

Supplement: Supplementary Figure 2 — Follow-up of 11 patients’ C-peptide. Visit 1: First C-peptide test due to elevated blood glucose during ICIs treatment. The interval between each visit varied among different patients. [file Image_2.tiff]
